# Cancer Clinical Trial Participation at the 1-Year Anniversary of the Outbreak of the COVID-19 Pandemic

**DOI:** 10.1001/jamanetworkopen.2021.18433

**Published:** 2021-07-29

**Authors:** Joseph M. Unger, Hong Xiao, Michael LeBlanc, Dawn L. Hershman, Charles D. Blanke

**Affiliations:** 1 SWOG Cancer Research Network Statistics and Data Management Center, Seattle, Washington; 2 Public Health Sciences Division, Fred Hutchinson Cancer Research Center, Seattle, Washington; 3 Department of Medicineand Epidemiology, Columbia University Medical Center, New York, New York; 4 SWOG Cancer Research Network Group Chair’s Office, Portland, Oregon; 5 Knight Cancer Institute, Oregon Health & Science University, Portland

## Abstract

**Importance:**

During the initial outbreak of the COVID-19 pandemic, cancer clinical trial participation decreased precipitously. Given the continued pandemic—especially the severe wave of new cases and deaths in winter 2020 to 2021—a vital question is whether trial enrollments have remained low or even worsened.

**Objective:**

To examine the experience of cancer clinical trial enrollment 1 year after the COVID-19 outbreak.

**Design, Setting, and Participants:**

This cohort study examines initial enrollments to treatment trials and cancer control and prevention (CCP) trials conducted by the SWOG Cancer Research Network between January 1, 2016, and February 28, 2021. Participants include patients enrolled in the trials.

**Exposures:**

Landmark time points reflecting the onset and the apex, respectively, of the initial COVID-19 wave (March 1 to April 25, 2020) and the winter 2020 to 2021 wave (October 4, 2020, to January 23, 2021).

**Main Outcomes and Measures:**

This study used interrupted time-series analysis to examine enrollments over time related to the COVID-19–derived exposure variables using negative-binomial regression. Relative risk (RR) estimates representing weekly enrollment changes compared with expected rates (had the pandemic not occurred) were derived. The numbers of enrollments lost during the pandemic were estimated.

**Results:**

Overall, 29 398 patients (mean [SD] age, 60.3 [13.2] years) were enrolled (24 034 before the pandemic and 5364 during the pandemic), with 9198 patients (31.3%) aged 65 years or older, 17 199 female patients (58.6%), 3039 Black patients (10.8%), and 2260 Hispanic patients (7.9%). Most enrollments (19 451 [66.2%]) were to treatment trials. During the initial COVID-19 wave, there was a 9.0% model-estimated weekly reduction in enrollments (RR, 0.91; 95% CI, 0.89-0.93; *P* < .001), with effects compounding each week. Enrollment recovered thereafter, but decreased again during the winter 2020 to 2021 wave, although by only 2.0% each week (RR, 0.98; 95% CI, 0.97-0.99; *P* < .001). Overall, during the pandemic, actual enrollments were 77.3% of expected enrollments (5364 of 6913 enrollments; 95% CI, 70.5%-85.0%; *P* < .001). Actual enrollments were 54.0% of expected enrollments for CCP trials (1421 of 2641 enrollments; 95% CI, 43.0%-67.0%; *P* < .001) and 91.0% of expected enrollments for treatment trials (3922 of 4304 enrollments; 95% CI, 81.0%-102.0%; *P* = .12).

**Conclusions and Relevance:**

In this cohort study, clinical trial enrollments decreased during the full year of the COVID-19 pandemic. Enrollment reductions were primarily to CCP trials, whereas, remarkably, there was not strong evidence of enrollment reductions to treatment trials. This finding suggests that clinical research rapidly adapted to the circumstances of enrolling and treating patients on protocols during the COVID-19 pandemic.

## Key Points

**Question:**

Did enrollment to cancer clinical trials in a federally funded cooperative group change during the first full year of the COVID-19 pandemic?

**Findings:**

By use of an interrupted time-series analysis of 29 398 clinical trial patients, this cohort study found a precipitous decrease in enrollments during the initial COVID-19 wave, but only a modest reduction during the winter 2020 to 2021 wave. Over the entire year, steep enrollment reductions were found for cancer control and prevention trials, whereas for treatment trials, enrollments were similar to expected rates.

**Meaning:**

These findings suggest that clinical trial research rapidly adapted to the circumstances of enrolling and treating patients on protocols during the COVID-19 pandemic.

## Teaser

This cohort study examines the experience of cancer clinical trial enrollment 1 year after the COVID-19 outbreak.

## Introduction

Enrollment to cancer clinical trials is vital to enable their rapid conduct and the discovery of new treatments for patients with cancer. During the outbreak of the COVID-19 pandemic in early 2020, trial enrollment decreased precipitously.^1,2^ Recognizing the challenges of recruiting patients to clinical trials during a pandemic, major federal agencies provided guidance to allow more flexibility with respect to recruitment and follow-up of patients in trials.^3,4^ For example, because of pandemic-related limitations on the ability of patients to travel, consent for trial participation could be done by telephone rather than in person.^4^ Given the continued pandemic, especially the severe wave of new cases and deaths in the fall and winter of 2020 to 2021, a vital question for researchers is whether clinical trial enrollment has remained low.

The answer to this question is critical because low clinical trial enrollment associated with COVID-19 can delay the completion of trials and the determination of whether new treatments are effective. Lower enrollment may also exacerbate existing accrual challenges for some studies, contributing to more trials closing because of poor accrual, which represents a substantial loss of scientific, institutional, and patient resources.^5,6,7^ Taken together, long-term reductions in cancer clinical trial enrollment associated with the COVID-19 pandemic would slow the development of new therapies for patients with cancer. In this cohort study, we examine the full 1-year experience of cancer clinical trial enrollment after the COVID-19 outbreak in the US.

## Methods

### Data

We examined initial enrollments to clinical trials conducted by the SWOG Cancer Research Network, a National Cancer Institute (NCI)–sponsored National Clinical Trials Network group and a member of the NCI’s Community Oncology Research Program. Enrollments to treatment trials and cancer control (including symptom management, survivorship, and cancer care delivery) and prevention (CCP) studies between January 1, 2016, and February 28, 2021, were included.

All trials were previously approved by an institutional review board; patients previously provided written informed consent at trial enrollment. The current study was approved by the Fred Hutchinson Cancer Research Center’s institutional review board. This study follows the Strengthening the Reporting of Observational Studies in Epidemiology (STROBE) reporting guidelines for cohort studies.

### Statistical Analysis

We examined patterns of enrollment over time using negative-binomial regression given a disperse variation structure. For analysis purposes, enrollments were aggregated by week. Using interrupted time-series analysis, we examined whether patterns of weekly total enrollments differed over time in conjunction with prespecified periods defined by key COVID-19 pandemic landmark times. To aid in the identification of exposure parameters, daily death totals attributable to COVID-19 for the US were obtained from the COVID-19 Tracking Project.^8^ To generate average trajectories over time and to identify the estimated apex of waves of COVID-19 deaths, a 14-day moving average was used and was plotted using local polynomial regression fitting (ie, loess).^9^ In part, on the basis of this evaluation, separate exposure indicator variables were used to define the intervals. The first interval was the initial COVID-19 wave, starting the week of March 1 to 7, 2020, corresponding to the first contemporaneously announced death in the US from COVID-19 (on February 29, 2020)^10^ and shortly before the date the World Health Organization declared COVID-19 a global pandemic on March 11, 2020,^11^ and ending the week of April 19 to 25, 2020, the estimated apex of daily national COVID-19 deaths during the first wave of the pandemic. The second interval was the initial recovery period, starting the week of April 26 to May 2 through September 27 to October 3, 2020, the estimated nadir of daily national COVID-19 death totals in the US before the new wave of COVID-19 cases and deaths during the winter of 2020 to 2021. The third interval was the winter 2020 to 2021 wave, starting the week of October 4 to 10, 2020, and lasting until the week of January 17 to 23, 2021, the estimated apex of the daily death counts related to the winter 2020 to 2021 wave.^8^ The period January 17 to 23, 2021, until the end of the study period was not separately described given the limited data on this secondary recovery period.

Comparisons between patterns of treatment trial enrollments and CCP trial enrollments were conducted using interaction tests. Initial registrations beginning in 2016 were used to enable regression modeling of annual temporal (ie, seasonal) variation in enrollments before the outbreak. Indicator variables for calendar month were used to account for this variation, especially a commonly occurring reduction in new enrollments during the winter holiday season (December and January), and to account for potential temporal autocorrelation. An indicator variable to adjust for whether trial enrollments occurred before or after January 1, 2018, was included to implicitly account for differences early in the period (2016-2017 vs 2018-2019) in the availability of larger, phase 2 to 3 or phase 3 trials (65 vs 77 trials). The model was parameterized such that the period from January 1, 2018, through February 28, 2020, represented the reference period for interpretation of the pandemic-related model regression estimates and represented the most proximal prepandemic period and, thus, the model-derived expected (or counterfactual) trajectories over time. Absolute enrollments lost because of the pandemic were calculated as the difference between the sum of model-fitted (ie, estimated) actual enrollment counts (referred to as actual enrollments) and the sum of expected counts under the forecasted (ie, counterfactual) model assuming the pandemic did not occur. Similarly, relative risk (RR) estimates were defined as the ratio of percentage weekly change in actual enrollment counts to percentage weekly change in expected counts. To generate 95% CIs for the pandemic-derived exposure variables, we simulated 10 000 predictions per week under each model (actual [fitted] and expected [counterfactual]) using the coefficients and covariance matrix of each model using a multivariate normal distribution.^12^ The 2.5th and 97.5th percentiles of the simulated values represented the 95% CI. Newey-West SEs with autocorrelation of up to 4 lags were used within our models to accommodate serial autocorrelation in residual errors.^13^

By use of the same modeling approach, we also examined the extent to which enrollment among key demographic groups of patients differed after the pandemic outbreak compared with expected trends. We estimated and plotted the relative change in enrollment during the pandemic within categories defined by age (<65 vs ≥65 years), sex (female vs male), race (Black vs other race, by participant self-report), and ethnicity (Hispanic vs not Hispanic, by participant self-report). Race and ethnicity were assessed in this study because of previously observed barriers in access to clinical trials for selected patient groups.^14^ To explore potential associations between trial enrollment patterns and regional differences in pandemic severity, we categorized states according to whether the excess death rate during the pandemic was greater than or equal to 20% higher than the expected rate according to historical norms^15^ and examined relative changes in enrollment during the pandemic according to these categories.

Enrollment during the 1-year pandemic was also evaluated according to institutional and study characteristics, including by study setting (community vs academic centers) for both treatment and CCP trials, by study phase (phase 3 vs other) for treatment trials, and by trial type (cancer control vs prevention studies) for CCP trials. Analyses were conducted in R statistical software version 4.0.2 (R Project for Statistical Computing) using data obtained March 1, 2021. χ^2^ tests were used to compare patient characteristics. A 2-sided *P* < .05 indicated statistical significance.

## Results

### Patient Characteristics

In total, 29 398 patients (mean [SD] age, 60.3 [13.2] years) were enrolled, including 24 034 (81.8%) before the pandemic and 5364 (18.2%) in the full-year period March 1, 2020, to February 28, 2021. Treatment trial enrollments accounted for 66.2% of the cohort (19 451 participants). Among treatment trials, the 4 most commonly occurring cancers in the US cancer population accounted for 56.1% of all enrollments (breast, 4026 enrollments; colorectal, 428 enrollments; lung, 5426 enrollments; prostate, 1033 enrollments). Overall, 9198 patients (31.3%) were aged 65 years or older, 17 199 (58.6%) were women, 3039 (10.8%) were Black, and 2260 (7.9%) were Hispanic (Table 1). In treatment trials, proportionally fewer female patients (45.2% vs 51.4%; χ^2^_1_ = 48.37; *P* < .001) and more Black patients (9.9% vs 8.5%; χ^2^_1_ = 7.40; *P* = .007) enrolled during the pandemic; in CCP trials, proportionally more female patients (83.8% vs 73.8%; χ^2^_1_ = 65.88; *P* < .001) and fewer Hispanic patients (5.3% vs 10.5%; χ^2^_1_ = 36.24; *P* < .001) enrolled during the pandemic. Enrollments from states with COVID-19–related excess death rates during the pandemic of greater than or equal to 20% were less likely for CCP trials (63.3% vs 68.7%; χ^2^_1_ = 16.48; *P* < .001), but no differences were observed with respect to enrollments to treatment trials (54.0% vs 54.2%; χ^2^_1_ = 0.09; *P* = .77).

**Table 1.** Patient Characteristics

Characteristics	Patients, No. (%)^a^			*P* value^b^
	All patients (N = 29 398)	Before pandemic (n = 24 034)	During pandemic (n = 5364)	
All studies (n = 29 398)				
Age, y				
<65	20 200 (68.7)	16 464 (68.5)	3736 (69.6)	.10
≥65	9198 (31.3)	7570 (31.5)	1628 (30.4)	
Sex				
Female	17 199 (58.6)	14 235 (59.3)	2964 (55.4)	<.001
Male	12 150 (41.4)	9765 (40.7)	2385 (44.6)	
Unknown	49	34	15	
Race				
Black	3039 (10.8)	2470 (10.7)	569 (11.2)	.34
Other	25 080 (89.2)	20 560 (89.3)	4520 (88.8)	
Missing or unknown	1279	1004	275	
Ethnicity				
Hispanic	2260 (7.9)	1899 (8.1)	361 (6.9)	.005
Non-Hispanic	26 390 (92.1)	21 553 (91.9)	4837 (93.1)	
Unknown	748	582	166	
State-level excess death rate during pandemic				
≥20%^c^	17 298 (58.8)	14 271 (59.4)	3027 (56.4)	<.001
<20%	12 100 (41.2)	9763 (40.6)	2337 (43.6)	
Treatment studies (n = 19 451)				
Age, y				
<65	12 932 (66.5)	10 379 (66.9)	2553 (64.7)	.007
≥65	6519 (33.5)	5124 (33.1)	1395 (35.3)	
Sex				
Female	9744 (50.1)	7967 (51.4)	1777 (45.2)	<.001
Male	9692 (49.9)	7536 (48.6)	2156 (54.8)	
Unknown	15	0	15	
Race				
Black	1637 (8.8)	1266 (8.5)	371 (9.9)	.007
Other	16 938 (91.2)	13 577 (91.5)	3361 (90.1)	
Missing or unknown	876	660	216	
Ethnicity				
Hispanic	1307 (6.9)	1019 (6.7)	288 (7.5)	.09
Non-Hispanic	17 617 (93.1)	14 079 (93.3)	3538 (2.5)	
Unknown	527	405	122	
State-level excess death rate during pandemic				
≥20%^c^	10 540 (54.2)	8409 (54.2)	2131 (54.0)	.77
<20%	8911 (45.8)	7094 (45.8)	1817 (46.0)	
CCP studies (n = 9947)				
Age, y				
<65	7268 (73.1)	6085 (71.3)	1183 (83.5)	<.001
≥65	2679 (26.9)	2446 (28.7)	233 (16.5)	
Sex				
Female	7455 (75.2)	6268 (73.8)	1187 (83.8)	<.001
Male	2458 (24.8)	2229 (26.2)	229 (16.2)	
Unknown	34	34	0	
Race				
Black	1402 (14.7)	1204 (14.7)	198 (14.6)	.91
Other	8142 (85.3)	6983 (85.3)	1159 (85.4)	
Missing or unknown	403	344	59	
Ethnicity				
Hispanic	953 (9.8)	880 (10.5)	73 (5.3)	<.001
Non-Hispanic	8773 (90.2)	7474 (89.5)	1299 (94.7)	
Unknown	221	177	44	
State-level excess death rate during pandemic				
≥20%^c^	6758 (67.9)	5862 (68.7)	896 (63.3)	<.001
<20%	3189 (32.1)	2669 (31.3)	520 (36.7)	

^a^ Missing and unknown data were not included in calculations of percentages.

^b^ *P* values were derived from χ^2^ tests.

^c^ In descending order of state-level excess death rates during the pandemic, this includes enrollments from any sites (excess death rate during pandemic) in New York (40%), New Jersey (34%), Arizona (31%), Mississippi (28%), Texas (26%), Connecticut (25%), Illinois (25%), North Dakota (24%), South Dakota (24%), Louisiana (23%), District of Columbia (23%), Wyoming (23%), Alabama (22%), California (22%), Michigan (22%), New Mexico (22%), South Carolina (22%), Arkansas (21%), Kansas (21%), Maryland (21%), Colorado (20%), Georgia (20%), Nevada (20%), and Rhode Island (20%).

### Pandemic Landmark Times and All Trial Enrollments

**Figure 1.**
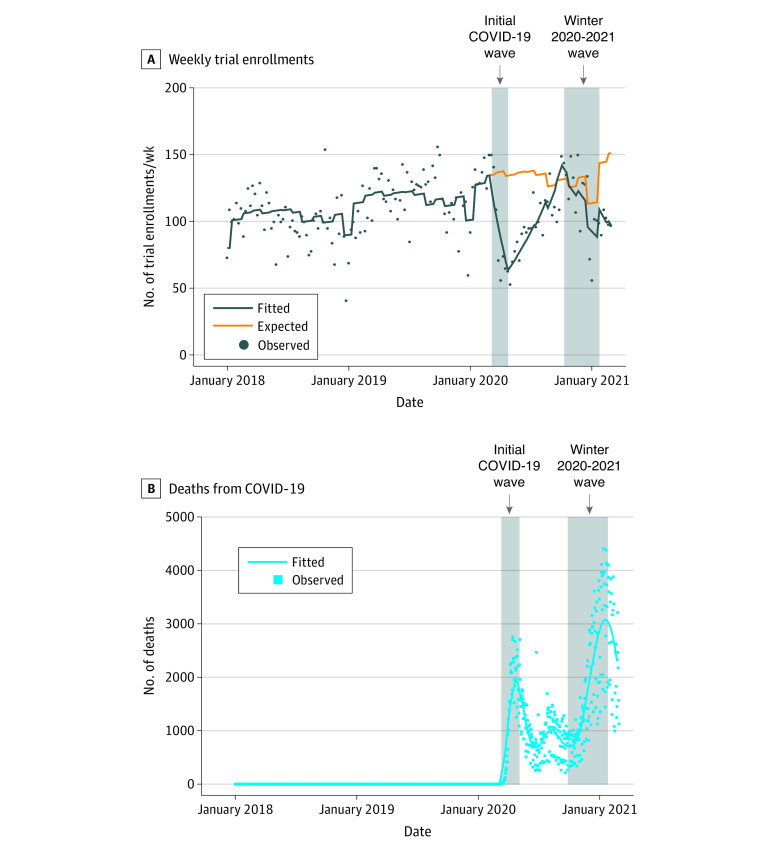
Trial Enrollments and Deaths From COVID-19 Over Time A, Weekly enrollments to National Cancer Institute–sponsored trials over time. Circles indicate actual (model-estimated) totals by week. The blue line shows the model fitted regression line for actual enrollments; the orange line represents model-estimated expected (ie, counterfactual) enrollments had the pandemic not occurred. The shaded areas illustrate the study-specified initial COVID-19 wave from March 1, 2020, through April 25, 2020, and the winter 2020 to 2021 COVID-19 wave from October 4, 2020, through the end of the analysis period, January 23, 2021. B, COVID-19 deaths over time. The squares show the daily totals, and the line shows a 14-day moving average. The shaded areas illustrate the same study-specified COVID-19 waves as shown in panel A. For both panels, the tick marks on the x-axes represent January 1 of each year.

As shown in Figure 1, the onset of the initial wave of COVID-19 deaths in the late winter of 2020 was associated with a substantial decrease in weekly trial enrollments. During the initial wave, from March 1, 2020, to April 25, 2020, there was a relative 9.0% model-estimated reduction per week in enrollments compared with the prepandemic period (RR, 0.91; 95% CI, 0.89-0.93; *P* < .001) (Table 2), reflecting a reduction in the total number of enrollments from an expected total of 1087 had the pandemic not occurred to an actual total of 730. For the week at the end of the initial COVID-19 wave, the expected number of enrollments was 134, compared with only 64 observed enrollments.

**Table 2.** Interrupted Time-Series Regression Model Estimates

Domain	Weekly change in enrollments vs expected rates had the pandemic not occurred			
	RR (95% CI)^a^	*P* value	RR ratio for treatment vs CCP (95% CI)	*P* value
Initial COVID-19 wave (week of February 24 to March 2, 2020, through week of April 12-18, 2020)				
All studies	0.91 (0.89-0.93)	<.001	0.90 (0.82-0.97)	.01
Treatment^b^	0.94 (0.92-0.95)	<.001		
CCP	0.84 (0.77-0.91)	<.001		
Initial recovery period (week of April 19-25, 2020, through week of September 27 to October 3, 2020)				
All studies	1.04 (1.03-1.05)	<.001	1.02 (0.98-1.05)	.37
Treatment^b^	1.03 (1.02-1.04)	<.001		
CCP	1.05 (1.02-1.09)	.006		
Winter 2020-2021 wave (week of October 4-10, 2020, through week of January 10-16, 2021)				
All studies	0.98 (0.97-0.99)	<.001	1.00 (0.97-1.02)	.74
Treatment^b^	0.98 (0.97-0.99)	.002		
CCP	0.97 (0.95-0.99)	.04		

Abbreviations: CCP, cancer control and prevention; RR, relative risk.

^a^ Indicates ratio of actual (fitted) weekly percentage change to expected (counterfactual) weekly percentage change.

^b^ Indicates reference category for comparing the ratio of RRs between treatment trials and CCP trials.

In contrast, the rate of enrollment per week increased substantially from April 26, 2020, to October 3, 2020. During this recovery period, a relative 4.0% model-estimated increase per week in enrollments compared with the prepandemic period was observed (RR, 1.04; 95% CI, 1.03-1.05; *P* < .001). Although the rate of change of enrollments per week was greater than during the prepandemic period, enrollment totals at the beginning of the recovery period were much lower; thus, during this initial recovery period, a total of 2295 actual enrollments were observed, compared with an expected total of 3079 had the pandemic not occurred. By the end of the recovery period, actual weekly enrollments (142 enrollments) exceeded expected enrollments (131 enrollments).

**Figure 2.**
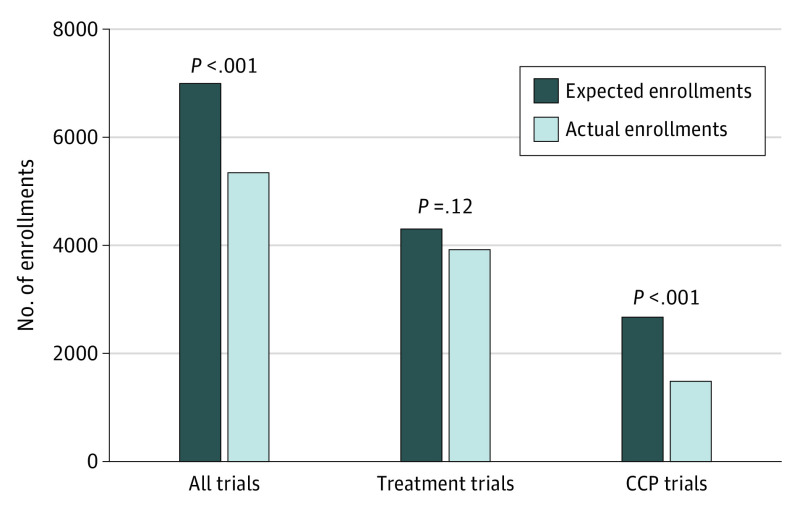
Expected vs Model-Fitted (Actual) Enrollments by Type of Trial CCP indicates cancer control and prevention.

During the winter 2020 to 2021 wave of COVID-19 deaths, enrollment again decreased. Some of this decrease was associated with seasonal variation, especially reductions in enrollments typically observed during each end-of-year holiday period. Accounting for this in the model, a relative 2.0% weekly reduction in enrollments compared with the prepandemic period was observed (RR, 0.98; 95% CI, 0.97-0.99; *P* < .001), even as the apex of the smoothed average number of daily COVID-19 deaths was notably higher (3077 deaths) compared with the apex of the initial COVID-19 wave (1931 deaths). During the entirety of the 1-year pandemic period, the model-estimated number of expected enrollments had the pandemic not occurred was 6913, compared with the actual total of 5344 (77.3% of expected; 95% CI, 70.5%-85.0%; *P* < .001), a difference of 1569 (22.7%) fewer patients (Figure 2).

### Pandemic Landmark Times and Treatment vs CCP Trial Enrollments

The weekly relative reduction in enrollments during the initial COVID-19 wave for CCP trials was 16.0% (RR, 0.84; 95% CI, 0.77-0.91; *P* < .001), more than twice as large as the 6.0% weekly relative reduction observed for treatment trials (RR, 0.94; 95% CI, 0.92-0.95; *P* < .001; ratio of RRs, 0.90; 95% CI, 0.82-0.97; *P* = .01) (Table 2). In contrast, during the winter 2020 to 2021 period, the weekly relative reduction in enrollments was only 3.0% (RR, 0.97; 95% CI, 0.95-0.99; *P* = .04) for CCP trials and only 2.0% (RR, = 0.98; 95% CI, 0.97-0.99; *P* = .002) for treatment trials. There was no statistically significant difference in the weekly reduction of enrollments between treatment and CCP trials (ratio of RRs, 1.00; 95% CI, 0.97-1.02; *P* = .74).

During the entirety of the 1-year pandemic period, the model-estimated number of expected enrollments had the pandemic not occurred was 4304 for treatment trials compared with an actual total of 3922 (91.0% of expected; 95% CI, 81.0%-102.0%; *P* = .12) (Figure 2). For CCP trials, the model-estimated number of expected enrollments had the pandemic not occurred was 2641 compared with an actual total of 1421 (54.0% of expected; 95% CI, 43.0%-67.0%; *P* < .001), a 46.0% reduction.

### Enrollments in Subgroups

**Figure 3.**
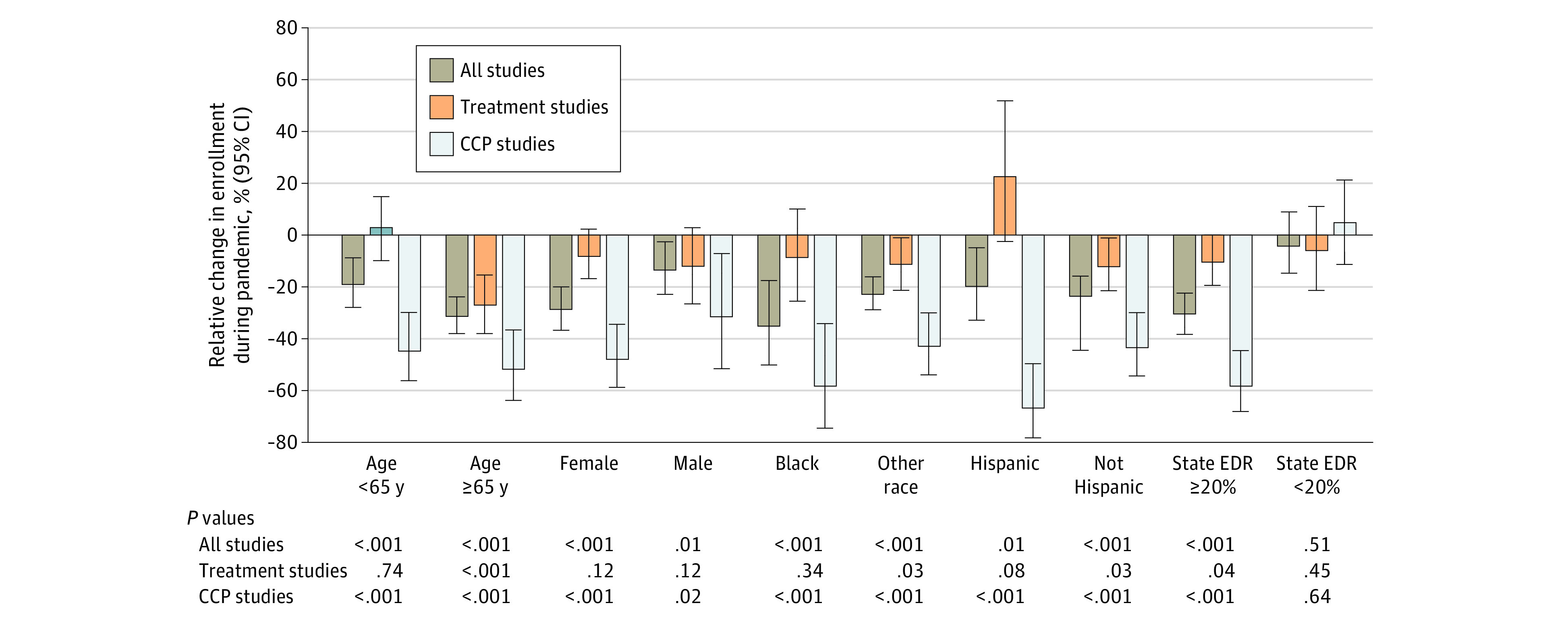
Relative Change in Enrollment During the Pandemic vs Expected Enrollment (Had the Pandemic Not Occurred) by Patient Groups Specific point estimates for relative change and the corresponding 95% CIs are provided in the eTable in the Supplement. CCP indicates cancer control and prevention; EDR, excess death rate.

The substantial reduction in enrollments to CCP trials during the initial COVID-19 wave is evident for all demographic subgroups (Figure 3 and eTable in the Supplement). In contrast, for treatment trials, there were proportionally fewer patients aged 65 years or older; otherwise, the relative reductions in enrollments during the pandemic were either not statistically significant or were only marginally statistically significantly different. For both treatment and CCP trials, there were proportionally fewer patients enrolled during the pandemic from states with higher COVID-19–related excess death rates; in contrast, there was no evidence of proportionally fewer patients enrolled from states with low COVID-19–related excess death rates during the pandemic.

The magnitude of the enrollment decrease was similar at community and academic sites for both treatment and CCP trials (eFigure in the Supplement). In treatment trials, enrollment to phase 3 studies was similar (2.0%; 95% CI, –15.8% to 22.3%; *P* = .87) during the pandemic, whereas enrollment to non–phase 3 trials decreased (–20.9%; 95% CI, –36.1% to –3.2%; *P* = .02). Among CCP trials, enrollment to prevention trials decreased more (–68.9%; 95% CI, –79.7% to –54.1%; *P* < .001) than enrollment to cancer control trials (–39.1%; 95% CI, –52.3% to –23.2%; *P* < .001).

## Discussion

In this study, we found that enrollment to NCI-sponsored cancer clinical trials decreased substantially during the initial outbreak of the COVID-19 pandemic. Thereafter, enrollment rebounded, even exceeding expected rates by the end of the prolonged recovery period before the winter 2020 to 2021 wave. The severe winter 2020 to 2021 wave was associated with yet another reduction in clinical trial enrollments, although this reduction was much more modest compared with the initial wave. Over the entire first year of the pandemic, an estimated 1569 (22.7%) fewer patients were enrolled because of the pandemic. Importantly, this enrollment reduction was predominantly observed for CCP trials (46.0% reduction) compared with treatment trials (9.0% reduction), with similar reductions observed at community and academic sites. Encouragingly, there was no evidence of a reduction in enrollments for Black or Hispanic patients to treatment trials over the entirety of the pandemic year. In contrast, reductions in enrollments to CCP trials were evident among all demographic subgroups of patients, especially for Black and Hispanic patients. Finally, enrollments for both CCP and treatment trials decreased substantially in states that experienced higher rates of excess deaths, but not in states with lower rates of excess deaths.

Oncologists have reported the intensive steps taken to enhance control of the physician-patient environment in order to limit opportunities for COVID-19 infection transmission during oncology clinic visits.^16^ Such strategies have been necessary because patients with cancer are likely more susceptible to the adverse consequences of COVID-19 given that their immune systems have been weakened by cancer and its treatments.^17,18,19^ Consistent with these strategies, key recommendations from federal guidance have included remote consent, use of virtual visits, increased flexibility for local health care practitioners to administer protocol therapy where possible, and allowance of alternative laboratories or imaging centers to conduct protocol-required tests and scans.^3,4^ Despite the initial decrease in enrollment associated with the initial outbreak of COVID-19, the strong rebound through the remainder of the first year of the outbreak suggests that institutions were successful in adapting new recruitment strategies to the circumstances of the pandemic. In this context, the goal has been to maintain patient safety while continuing the forward momentum of clinical cancer research.^16^

Social inequalities in both outcomes from disease and access to health care resources for COVID-19 have been clearly demonstrated.^20,21^ A particular concern in cancer is that existing inequities in trial participation by socioeconomic and demographic characteristics may be exacerbated.^14,22^ For treatment trials, we found consistent enrollment for Black and Hispanic patients over the full year of the pandemic. In contrast, we found a notable reduction in enrollments for both Black and Hispanic patients to CCP trials during the first full year of the pandemic. For Black patients, these reductions were consistent with reductions for non-Black patients, whereas for Hispanic patients, reductions were much greater than for non-Hispanic patients. These patterns may reflect differential challenges for this group of patients with respect to employment, access to health care resources, and language that are heightened during the pandemic with respect to participation in trials with less immediate impact on survival outcomes.^23,24^ Furthermore, although enrollment to CCP trials was lower for both sexes, the reduction in enrollment of female patients was notably larger, potentially reflecting higher job loss or increased responsibility caring for children.^25,26^

### Strengths and Limitations

A strength of this study is that it included findings from nearly 30 000 patient enrollments to a diverse set of cancer treatment and CCP trials. Moreover, we used a time-series analysis that was necessary to account for underlying seasonal trends in enrollment that could otherwise either mask or accentuate the impact of the COVID-19 outbreak.

However, this study was limited by the inclusion of a single NCI-sponsored national network group, so the results may not generalize to all clinical cancer research settings. In addition, although we adjusted for the number of accruing trials over time, differences in the types of trials available could still account for some differences in the observed patterns. This latter consideration is further complicated by the observation that the COVID-19 pandemic has also been associated with reduced activation of new trials^27^; thus, more detailed adjustment for this factor in the model could end up understating the impact of the pandemic on trial enrollments. Furthermore, information was not available about whether specific institutions paused enrollment to trials during the pandemic, which could aid interpretation.

## Conclusions

The health impacts of the COVID-19 pandemic have been far reaching, extending beyond morbidity and mortality from COVID-19 itself. Both domestically and internationally, researchers have shown that COVID-19 is associated with reduced opportunities for screening, treatment, and survivorship care for common diseases, including stroke, cancer, and cardiovascular disease.^18,28,29,30,31,32^ The activation of new clinical research studies has also been delayed.^27,33^ Our findings add another dimension to these investigations by demonstrating how large reductions in cancer clinical trial enrollments occurred during the initial pandemic wave. Thus, care for existing patients with cancer and research into new treatments for future patients has been adversely influenced by the pandemic.

Our findings also show how enrollment to clinical trials rebounded strongly after the initial wave of the pandemic. Although enrollment decreased again during the most severe COVID-19 wave of the winter of 2020 to 2021, the reduction was fairly modest. Remarkably, over the full year of the pandemic, there was no strong evidence of a reduction in enrollments to treatment trials. This may have occurred at the expense of full support of CCP trials, for which enrollment over the entire pandemic year was much lower. If so, this pattern could reflect institutional prioritization of resources to target the conduct of trials most directly related to patient care and also could reflect that clinical research has rapidly adapted to the circumstances of enrolling and treating patients on protocols during the COVID-19 pandemic. Future studies should be conducted to evaluate the quality of data collection and the potential influence on clinical outcomes in patients with cancer enrolled in trials during the pandemic.

## Notes

Supplement. **eTable.** Point Estimates and 95% Confidence Intervals (CI) for Relative Change in Enrollment During the Pandemic Within Patient Groups **eFigure.** Relative Change in Enrollment During the COVID-19 Pandemic Within Patient Groups
