# Supplementary material for: Cancer Clinical Trial Participation at the 1-Year Anniversary of the Outbreak of the COVID-19 Pandemic
Source: JAMA Netw Open. 2021 Jul 29;4(7):e2118433. doi: 10.1001/jamanetworkopen.2021.18433 (PMC8323000; doi:10.1001/jamanetworkopen.2021.18433)
Supplement: Supplement. — eTable. Point Estimates and 95% Confidence Intervals (CI) for Relative Change in Enrollment During the Pandemic Within Patient Groups eFigure. Relative Change in Enrollment During the COVID-19 Pandemic Within Patient Groups [file jamanetwopen-e2118433-s001.pdf]

Supplementary Online Content

Unger JM, Xiao H, LeBlanc M, Hershman DL, Blanke CD. Cancer clinical trial participation at the 1-year anniversary of the outbreak of the COVID-19 pandemic. *JAMA Netw Open*. 2021;4(7):e2118433.
doi:10.1001/jamanetworkopen.2021.18433

eTable. Point Estimates and 95% Confidence Intervals (CI) for Relative Change in Enrollment During the Pandemic Within Patient Groups

eFigure. Relative Change in Enrollment During the COVID-19 Pandemic Within Patient Groups

This supplementary material has been provided by the authors to give readers additional information about their work.

© 2021 Unger JM et al. *JAMA Network Open*.

eTable. Point Estimates and 95% Confidence Intervals (CI) for Relative Change in Enrollment During the Pandemic Within Patient Groups

**Characteristic**	**Category**	**Study set**	**Relative change (95% CI)**	**p-value**
Age	<65 years	All studies	-18.5 (-27.6 to -8.7)	<.001
		Treatment studies	2.3 (-9.6 to 14.7)	.74
		CCP studies	-44.4 (-56.2 to -29.9)	<.001
	65 or older	All studies	-31.4 (-38.0 to -24.0)	<.001
		Treatment studies	-27.1 (-37.6 to -15.4)	<.001
		CCP studies	-51.6 (-63.7 to -36.6)	<.001
Sex	Female	All studies	-28.6 (-36.7 to -20.0)	<.001
		Treatment studies	-7.7 (-16.8 to 2.1)	.12
		CCP studies	-47.8 (-58.6 to -34.5)	<.001
	Male	All studies	-13.3 (-22.7 to -2.8)	.01
		Treatment studies	-12.1 (-25.9 to 3.4)	.12
		CCP studies	-32.0 (-51.7 to -7.2)	.02
Race	Black	All studies	-35.6 (-50.2 to -17.7)	<.001
		Treatment studies	-8.8 (-25.5 to 10.1)	.34
		CCP studies	-58.3 (-74.5 to -34.4)	<.001
	Other	All studies	-22.6 (-28.8 to -15.9)	<.001
		Treatment studies	-11.5 (-21.2 to -1.0)	.03
		CCP studies	-43.0 (-54.1 to -30.0)	<.001
Ethnicity	Hispanic	All studies	-19.8 (-32.9 to -5.1)	.01
		Treatment studies	22.6 (-2.3 to 51.8)	.08
		CCP studies	-66.8 (-78.4 to -49.8)	<.001
	Not Hispanic	All studies	-23.5 (-30.6 to -15.9)	<.001
		Treatment studies	-11.7 (-21.4 to -1.0)	.03
		CCP studies	-43.0 (-54.4 to -30.0)	<.001
Excess death rate	20% or greater	All studies	-30.6 (-38.3 to -22.3)	<.001
		Treatment studies	-10.4 (-19.5 to -0.5)	.04
		CCP studies	-57.6 (-68.2 to -44.6)	<.001
	<20%	All studies	-3.8 (-14.8 to 9.0)	.51
		Treatment studies	-6.1 (-21.4 to 11.1)	.45
		CCP studies	4.3 (-11.3 to 21.3)	.64

CCP = cancer control and prevention

© 2021 Unger JM et al. *JAMA Network Open*.

eFigure. Relative Change in Enrollment During the COVID-19 Pandemic Within Patient Groups

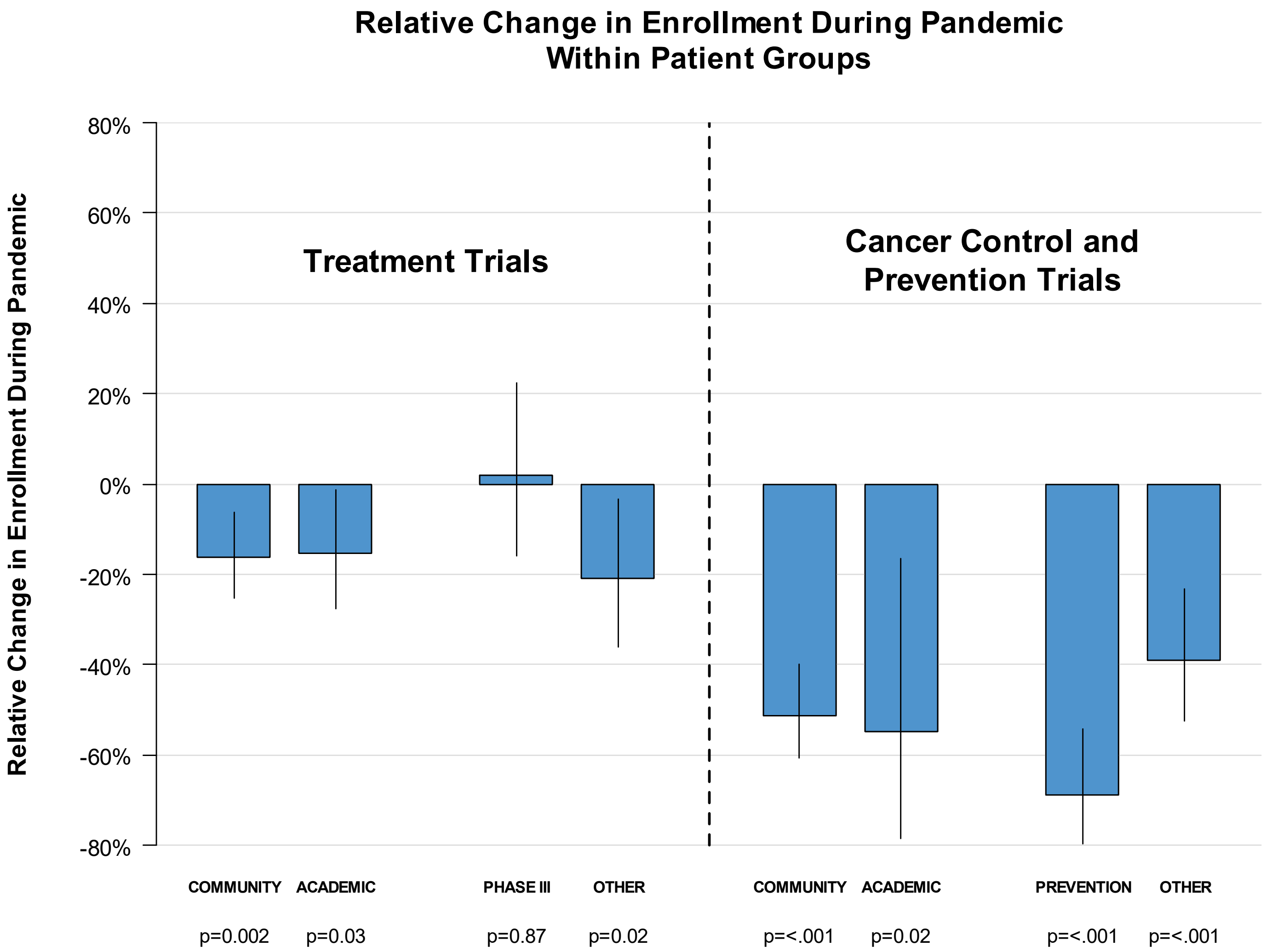

© 2021 Unger JM et al. *JAMA Network Open.*
